# Ultrasonication-mediated multi-micronutrient fortification of polished rice to address micronutrient deficiencies

**DOI:** 10.1016/j.ultsonch.2026.107814

**Published:** 2026-03-10

**Authors:** Glenn Vincent P. Ong, Iegem Lean Laudencia, Rhowell Tiozon, Jhaymes Khylle Jose, Vipin Pratap, Nese Sreenivasulu

**Affiliations:** a Consumer-driven Grain Quality and Nutrition Center, International Rice Research Institute, Los Baños, Philippines; b Centre of Excellence in Rice Value Addition (CERVA), International Rice Research Institute (IRRI) South Asia Regional Centre (ISARC), Varanasi, Uttar Pradesh, India

**Keywords:** Rice fortification, Ultrasonication, Micronutrient enrichment, B-vitamins, Mineral bioaccessibility, Food fortification

## Abstract

Ultrasonication-mediated multi-micronutrient fortification of polished rice was evaluated as a rapid and efficient strategy to enhance micronutrient loading and bioaccessibility. A one-pot process integrating soaking and sonication was optimized using response surface methodology (RSM), yielding optimal conditions of 2.73 min sonication, 5000 ppm fortificant concentration, and 240 min soaking time. Under these conditions, fortified rice showed markedly higher micronutrient concentrations than non-fortified controls, including iron (2802.42 ± 31.25 µg/g; ∼407-fold increase), zinc (813.05 ± 14.25 µg/g; ∼61-fold), thiamine (371.98 ± 13.92 µg/g; ∼413-fold), and folic acid (15.73 ± 0.32 µg/g; ∼8.4-fold). Simulated gastrointestinal digestion revealed nutrient-specific release patterns. Fortified rice retained high intestinal bioaccessibility of thiamine (78.93%), whereas the non-fortified sample showed complete loss at the intestinal phase. In contrast, folic acid exhibited reduced intestinal bioaccessibility (39.24%), indicating lower stability during digestion. Most minerals maintained measurable intestinal recovery, particularly iron (100.00%) and zinc (35.34%), indicating sustained solubility under simulated gastrointestinal conditions. Ultrasound treatment also altered physicochemical properties of the rice matrix. Rapid Visco Analyzer analysis showed significant reductions in peak viscosity (3463 → 1953 cP) and setback viscosity (3190 → 485 cP), indicating partial disruption of starch structure and reduced retrogradation.Texture profile analysis revealed decreased chewiness, while color measurements showed increased lightness in fortified samples. Overall, ultrasonication represents a scalable and energy-efficient approach for producing nutrient-dense rice kernels suitable for blending or food formulation, supporting scalable food fortification strategies to mitigate micronutrient deficiencies in rice-consuming populations.

## 1 Introduction

Micronutrient deficiencies affect more than 2 billion people globally, often referred to as hidden hunger, represent a major form of malnutrition arising from inadequate intake, poor absorption, or limited bioavailability of essential micronutrients such as iron, zinc, and vitamins. These deficiencies contribute to a wide range of adverse health outcomes, including anemia, impaired cognitive development, weakened immune function, and increased susceptibility to infections [1]. Recent global projections indicate that the global burden of disease associated with chronic and hidden hunger resulting from micronutrient deficiencies, expressed in disability-adjusted life years (DALYs), is expected to rise further and may increase by over 30 million DALYs by 2050 [2]. Most individuals affected by micronutrient deficiencies live in low- and middle-income countries, where limited dietary diversity increases reliance on staple crops.

Nearly 20% of the world’s caloric intake comes from rice, making it a primary food source for more than three billion people [3]. Yet despite its central role in global nutrition, milled rice—the form most widely consumed—is naturally low in essential micronutrients such as iron, zinc, and B group vitamins [4], [5]. As a result, rice-dependent populations remain vulnerable to micronutrient deficiencies that contribute to impaired growth, reduced cognitive development, increased morbidity, and long-term disability. Fortification strategies have been widely recognized as cost-effective public health interventions, with folic acid fortification alone estimated to cost approximately US$14.90 per disability-adjusted life year (DALY) averted and ∼US$957 per death averted in developing-country settings [6], [7]. In addition to improving health outcomes, rice fortification and related micronutrient interventions generate significant economic benefits through reduced healthcare expenditures and productivity losses linked to micronutrient deficiency-related disabilities.

Fortification of rice offers a scalable and cost-effective solution to address this nutritional gap, providing an efficient way to deliver essential micronutrients to at-risk populations. As a result, researchers and policymakers are increasingly prioritizing rice fortification and advanced processing technologies as key strategies to improve public health outcomes [8], [9]. Numerous regions have seen notable improvements in nutritional outcomes as a result of rice fortification. A classic example of how fortified staple foods can improve population health at a low cost is Costa Rica's mandatory rice fortification program, which incorporates iron, folic acid, and B group vitamins. This program has significantly decreased the prevalence of anemia and neural tube defects [10]. The World Food Programme and government-led initiatives in India and other countries in Asia and the Pacific have also highlighted the potential of fortified rice and wheat to prevent hunger and malnutrition. Regional organizations like the Association of South East Asian Nations are fostering progress by exchanging knowledge and standardizing practices [11]. Traditional rice fortification approaches have been widely applied in the food industry, including coating, dusting, soaking and parboiling, acid parboiling, cross-linking, and extrusion-based technologies [7]. Among these methods, extrusion-based fortification is currently one of the most widely implemented technologies; however, its adoption remains limited in low-resource settings because it requires substantial infrastructure investment. This highlights the need for high-throughput and cost-effective fortification technologies that enhance nutrient penetration and retention while preserving rice physicochemical quality and consumer acceptability [12].

Previously, we have shown how a quick and efficient ultrasonication process has successfully fortified rice with individual micronutrients such as iron and folic acid on a standalone basis. These ultrasonication high-frequency sound waves create acoustic cavitation, with microstructural disruption through the quick formation and collapse of microscopic bubbles that result in localized high pressures and temperatures [13]. This mechanism has been exploited in earlier studies to enhance the adsorption and uptake of water-soluble micronutrients in rice, including B-group vitamins such as pantothenic acid (vitamin B5), where sonicated milled rice exhibited a markedly higher vitamin uptake capacity compared with non-sonicated rice kernels [14]. Similarly, ultrasonic treatment has been shown to enable highly efficient folic acid absorption into the rice endosperm, with enriched levels reaching up to a 4,054-fold increase in milled rice [15], attributed to increased kernel porosity and microstructural fissuring induced by sonication. Compared with conventional fortification methods, ultrasonication enhances hydration kinetics, promotes uniform gelatinization, and facilitates deeper penetration of nutrient solutions into the endosperm. As a rapid, solvent-free, and energy-efficient process aligned with green chemistry principles, ultrasonication represents a promising and sustainable technology for producing nutritionally enhanced rice with improved functional and sensory qualities [13]. However, ultrasonication-assisted multi-micronutrient fortification and its impact on nutrient bioaccessibility have not yet been systematically investigated.

**Fig. 1:**
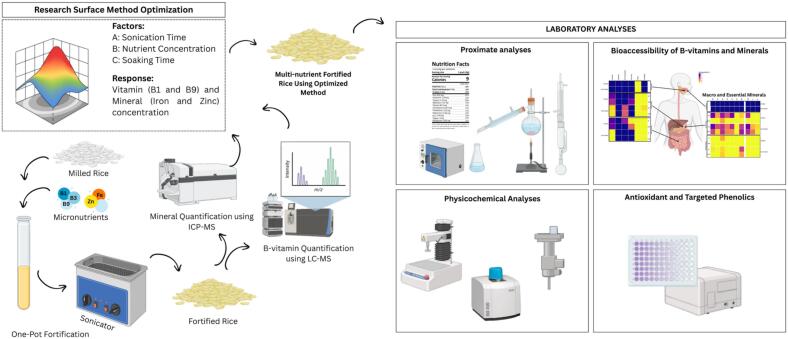
Schematic diagram of the ultrasound-assisted fortification of a milled rice sample using a one-pot fortification method.

This study hypothesizes that ultrasonication increases rice kernel porosity, thereby enhancing the uptake, stability, and retention of multiple micronutrients as an integrated approach to address deficiencies in iron, zinc, and B group vitamins. The objectives are to (i) evaluate the feasibility of ultrasonic-assisted incorporation of iron, zinc, and B group vitamins into rice, (ii) assess resulting changes in nutrient content and bioaccessibility, and (iii) determine the effects of sonication and micronutrient uptake on key quality attributes, including phenolic content, pasting behavior, and texture (Fig. 1). Overall, ultrasonication-assisted fortification is proposed as a promising strategy for producing nutritionally enhanced rice while maintaining desirable physicochemical and sensory-related properties.

## 2 Methodology

### 2.1 Rice sample and fortification method

Milled rice samples of IR 122297-B-5157-1-1 RGA-B (high-amylose and high-protein variety HAHP 108; 23.20% amylose and 9.28% dry basis crude protein), produced at the International Rice Research Institute (IRRI), Los Baños, Laguna, Philippines (2024 dry season), were used in this study. A one-pot fortification approach was applied in triplicate. Multi-micronutrient solutions were prepared by dissolving folic acid (Sigma Life Science F7876), ferrous sulfate heptahydrate (Merck Sigma-Aldrich F7002), zinc sulfate heptahydrate (Sigma-Aldrich 221376, ACS reagent ≥99%), niacinamide (Sigma-Aldrich N5535, USP specification), and thiamine hydrochloride (Sigma-Aldrich T4625, reagent grade ≥99% HPLC) in distilled water at three concentration levels (1000, 3000, and 5000 ppm), with each micronutrient individually present at the indicated concentration in the combined solution. Prior to sonication, rice grains were soaked in the multi-nutrient solution for 30, 135, or 240 min, according to the experimental design. Ultrasonication was performed using an Elmasonic EASY 60H ultrasonic bath operating at 37 kHz. Process parameters were optimized using response surface methodology (RSM), and the validated optimal conditions were 2.734 min sonication, 4999.99 ppm fortificant concentration, and 240 min soaking time. These optimized parameters were used for subsequent validation experiments and analyses. Following fortification, the kernels were dried in a hot-air oven at 50°C for approximately 4 h until moisture equilibrium was achieved. This mild drying condition was selected to minimize excessive thermal stress and structural damage while ensuring adequate removal of surface moisture prior to storage and analysis. Unless otherwise specified, both fortified and non-fortified rice samples were cooked using a standardized water-to-rice ratio of 1.4:1 (w/w) to simulate typical household preparation. All nutrient, physicochemical, and bioaccessibility analyses were conducted on cooked samples to reflect realistic consumption conditions.

### 2.2 Response surface methodology

Optimization of fortification parameters was performed using Box-Behnken Design (BBD) to develop robust quadratic models for reliable prediction within the experimental domain similar to the previous study [16]. Three independent variables were investigated: sonication time (0–15 min), nutrient concentration (1000–5000 ppm), and soaking time (30–240 min). The response variables measured were thiamine (Vitamin B1), folic acid (Vitamin B9), iron, and zinc content in fortified rice samples. A total of 17 experimental runs were conducted according to the BBD matrix. The desirability function was employed to simultaneously maximize the retention of thiamine, folic acid, iron, and zinc while maintaining the process variables within the specified ranges. This approach was used to determine the optimal fortification conditions. The solution meeting the desirable optimization criteria was selected for subsequent fortification experiments and analyses.

The relationship between response variables and independent factors was described using second-order polynomial equations:

(1) $$Y=b_{0}+b_{\text{1}}A+b_{2}B+b_{\text{3}}C+b_{\text{4}}AB+b_{\text{5}}AC+b_{\text{6}}BC+b_{\text{7}}A^{2}+b_{\text{8}}B^{2}+b_{\text{9}}C^{2}$$

where *Y* is the predicted response, *b*_0_ is the constant coefficient, *b*_1_–*b*_9_ are the linear, interaction, and quadratic coefficients, and *A*, *B*, and *C* represent sonication time, nutrient concentration, and soaking time, respectively. The statistical significance of model coefficients was evaluated at a 95% confidence level using Design-Expert software (Stat-Ease Inc., Minneapolis, MN, USA). Confirmatory experiments were conducted to validate the predicted optimal conditions.

### 2.3 B-vitamins quantification analyses

Quantification of B group vitamins (thiamine, pyridoxine, pantothenic acid, folic acid, biotin, and riboflavin) was performed in triplicates using an Agilent 6470 Triple Quad LC-MS/MS system by following the methodology of Itagi et al. (2025) [17]. The extraction procedure involved sample homogenization in pH 7.0 phosphate buffer, followed by sequential enzymatic treatment with α-amylase and protease to release vitamins from their bound forms. After centrifugation, the samples were filtered using nylon filters and subsequently analyzed by LC-MS/MS (Agilent 6470 Triple Quad). Method validation, including linearity assessment and calibration, was conducted using NIST-certified SRM 1869.

### 2.4 Mineral content quantification analyses

Essential minerals (magnesium, potassium, phosphorus, manganese, iron, cobalt, copper, and zinc) and heavy metals (arsenic, tin, and cadmium) were quantified using inductively coupled plasma-mass spectrometry (ICP-MS, Agilent 7800) in triplicate [17], [18]. Samples were digested via microwave-assisted acid digestion with concentrated nitric acid at 180°C to ensure complete mineralization. Following cooling and dilution with ultrapure water, digests were filtered through PVDF membranes prior to ICP-MS analysis. Helium collision mode was employed for simultaneous multi-element quantification at trace concentrations while reducing polyatomic interferences. Recovery and accuracy were verified using NIST SRM 1568b with each analytical batch.

### 2.5 Targeted phenolic profile

Targeted analysis of phenolic compounds was conducted using LC-MS/MS following phenolic extraction from defatted rice samples as per Jayaraman et al. (2019) [19]. Twelve specific phenolic compounds were quantified: kaempferol (C_15_H_10_O_6_), apigenin (C_15_H_10_O_5_), luteolin (C_15_H_10_O_6_), 4-hydroxybenzoic acid (C_7_H_6_O_3_), epicatechin (C_15_H_14_O_6_), sinapic acid (C_11_H_12_O_5_), p-coumaric acid (C_9_H_8_O_3_), caffeic acid (C_9_H_8_O_4_), quercetin (C_15_H_10_O_7_), *trans*-ferulic acid (C_10_H_10_O_4_), syringic acid (C_9_H_10_O_5_), and vitexin (C_21_H_20_O_10_).

### 2.6 Proximate analyses

Proximate analysis was conducted according to the procedures outlined by Itagi et al. (2025) [17]. The Kjeldahl nitrogen method was employed for protein determination using a Kjeltec™ 8200 system (Foss, Sweden), while lipid content was measured via Soxhlet extraction (Foss ST243, Foss, Sweden) utilizing petroleum ether (boiling range of 40-60°C). Ash content was obtained through incineration of samples in a muffle furnace at 550°C. Total carbohydrate was determined by difference, calculated by subtracting moisture, protein, ash, and fat values from 100, with results reported as g/100 g. Energy content (kJ/100 g dry matter) was estimated using the Atwater factors described by Eknayake et al. (1999) [20]: Energy (kJ/100 g) = (Protein × 16.7) + (Fat × 37.7) + (Total Carbohydrate × 16.7) (2). The calculated energy values in kilojoules were converted to kilocalories using the conversion factor of 1 kJ = 0.239006 kcal.

### 2.7 Color parameters

Colorimetric analysis was performed on fortified and non-fortified rice samples using a Konica Minolta Chroma Meter CR-400. The instrument quantified color parameters (L*, a*, b*, chroma, and hue) based on the CIELAB color space system. In this system, L* denotes lightness on a scale from 0 (black) to 100 (white), a* indicates chromaticity along the red-green axis (where positive values represent red and negative values represent green), and b* indicates chromaticity along the yellow-blue axis (where positive values represent yellow and negative values represent blue).

### 2.8 Texture profile analyses

Texture analysis was conducted on twenty-five whole cooked milled rice grains per accession following a modified version of Misra et al. (2018) [21]. Grains were washed three times and soaked in 1 mL of Milli-Q water for 30 min within test tubes. Following hydration, samples underwent heating to boil for 20 min, then held at 50°C to inhibit starch retrogradation prior to instrumental analysis. A Ta.XT-Plus Texture Analyzer (Stable Micro Systems Ltd., Surrey, UK) equipped with a 35-mm diameter aluminum cylindrical probe and 5-kg load cell was utilized for texture profiling. The probe was set 15 mm above the base platform, and three parallel intact cooked kernels were positioned centrally beneath it on an aluminum plate. Samples were subjected to two-cycle compression at 90% strain with both test and post-test velocities set at 0.5 mm s^−1^. From the generated force–deformation profiles, the following textural attributes were calculated: hardness (HRD: maximum force during initial compression normalized by curve height), adhesiveness (ADH: area of negative force during first bite cycle, expressed in absolute terms), cohesiveness (COH: ratio of second to first compression area, A2/A1), and springiness (SPR: ratio of time periods, T2/T1). Data acquisition and processing were performed using Exponent Lite Software (version 3.0.5.0). All texture measurements included three technical replicates across three biological replicates.

### 2.9 Gelatinization properties – rapid visco analyzer

Starch pasting properties were analyzed using a Perkin Elmer RVA 4800 (Rapid Visco Analyzer) in accordance with AACC method 61-02. The instrument monitored viscosity changes during a programmed temperature profile that began at 50°C, ramped to 95°C, maintained at this temperature, and subsequently cooled to 50°C.

### 2.10 *In vitro* bioaccessibility of minerals and B-vitamins

The bioaccessibility of minerals and B-vitamins in the cooked fortified and non-fortified rice samples was evaluated using a static in vitro digestion model comprising three sequential phases (oral, gastric, and intestinal) following the INFOGEST protocol [16], [22]. This method simulates human digestion using stage-specific enzymes and simulated digestive fluids. Cooked rice samples (5 g) were subjected to digestion using a standardized 1:1 ratio with simulated salivary fluid (5 mL) during the oral phase prior to sequential gastric and intestinal digestion steps. Sample aliquots (0.5 mL) were withdrawn at defined time points during the gastric phase (G0 at 0 min and G120 at 120 min) and intestinal phase (I0 at 0 min and I120 at 120 min). After centrifugation (9000*g*, 10 min), the supernatant representing the solubilized (potentially bioaccessible) fraction was collected. The bioaccessibility percentage for each nutrient was calculated according to De Meneses Costa Ferreira et al. (2022) [23] as follows:

(3) $$\%\text{Bioaccessibility}\;=\left(\text{Concentration in soluble fraction after digestion}\;/\;\text{Initial concentration in cooked sample before digestion}\right)\times \text{1}00$$

### 2.11 Starch properties

Starch digestibility, digestible carbohydrates, and resistant starch were quantified enzymatically using a Megazyme assay kit (K-RSTAR, Megazyme, Ireland). Amylose and amylopectin fractions were determined using high-performance liquid chromatography–size exclusion chromatography (HPLC–SEC), and the amylose/amylopectin ratio was calculated from the quantified fractions.

### 2.12 Statistical analysis

Data are expressed as mean ± standard deviation from three replicates. Significant differences among groups were determined using one-way analysis of variance (ANOVA) with Tukey's Honestly Significant Difference (HSD) post-hoc test at α = 0.05 using Minitab Statistical Software. Pearson's correlation analysis was performed to evaluate associations among physical properties, textural attributes, nutritional composition, bioactive compounds, and antioxidant activity. Graphical representations were generated using R software version 4.2.1 (Vienna, Austria).

## 3 Results and discussion

### 3.1 Optimization of fortification parameters using response surface methodology

A Box–Behnken design with three variables—sonication time (0–15 min), nutrient concentration (1000–5000 ppm), and soaking time (30–240 min)—was used to optimize ultrasound-assisted multi-nutrient rice fortification across 17 experimental runs (Supplementary Table 1). Quadratic response surface models showed an excellent fit for experimental data. All nutrient models were highly significant (p < 0.001), with high R^2^ values (0.9502–0.9905) and closely aligned adjusted R^2^ values (0.9365–0.9810), confirming strong model adequacy without overfitting (Supplementary Table 2). Adequate precision ratios (>13) indicated robust signal-to-noise performance suitable for design space navigation. Low coefficients of variation (c.v.) for thiamine, iron, and zinc (5.17–8.77%) reflected good reproducibility, whereas the higher c.v. for folic acid (43.47%) likely reflects its inherent chemical instability and variable mass-transfer behavior. Lack-of-fit was non-significant for thiamine and zinc and acceptable for folic acid and iron due to their high explanatory power (Supplementary Table 3). The constraints and numerical output for the optimization of the fortification process and the equations coded factors for different responses (Supplementary Tables 3 and 4). Moreover, regression coefficients revealed nutrient concentration as the most influential positive factor across all nutrients, while sonication time showed contrasting effects, with significantly reduced thiamine retention but had U-shaped quadratic effects on iron and zinc, indicating benefits at moderate exposure but degradation at extended durations. Soaking time consistently improved retention of thiamine, iron, and zinc by enhancing diffusion. Significant interaction effects—especially between sonication and concentration—demonstrated that factor effects were interdependent, reinforcing the non-linear behavior confirmed by significant quadratic terms.

Among all treatments, the highest levels of thiamine (1888.53 ± 28.57 μg/g), iron (3134.61 ± 18.93 μg/g), and zinc (937.96 ± 20.21 μg/g) occurred in Treatment 8 (0 min sonication, 5000 ppm, 135 min soaking), suggesting that the absence of ultrasound protected heat-labile vitamins from cavitation-induced degradation. In contrast, folic acid reached its maximum (186.98 ± 15.72 μg/g) under moderate sonication (Treatment 3 = 7.5 min sonication, 1000 ppm, 30 min soaking), and the lack of significant differences among several treatments suggests lower sensitivity or earlier saturation of its uptake. Since folic acid is a vital micronutrient, optimization using the desirability function identified 2.734 min sonication, 4999.999 ppm concentration, and 240 min soaking (D = 0.542) as the optimal conditions for all the micronutrients, yielding balanced improvements across all nutrients (Supplementary Table 5).

### 3.2 Optimized fortification revealed improvements in rice nutritional quality

**Fig. 2:**
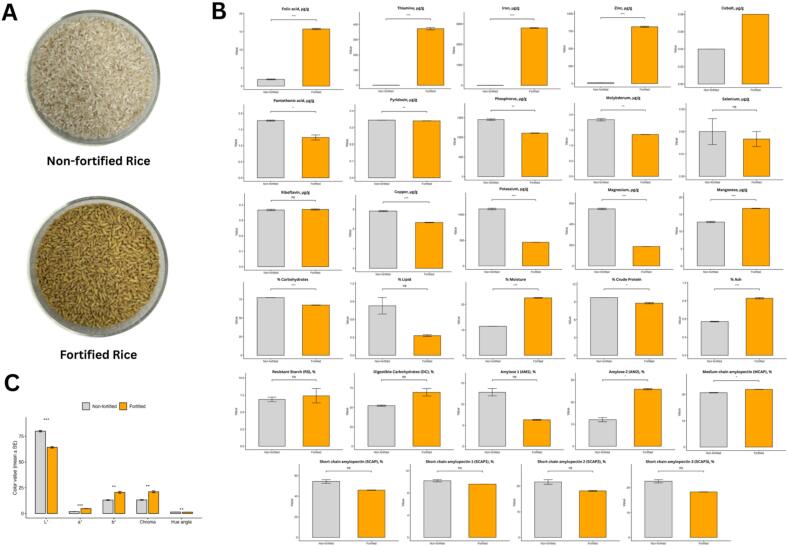
Comparison of non-fortified and fortified rice produced under optimized ultrasound-assisted conditions. (A) Visual appearance showing the physical appearance of non-fortified and fortified rice. (B) Differences in proximate composition, B-vitamins, minerals, and starch-related functional parameters (moisture, ash, protein, lipids, carbohydrates, folic acid, pantothenic acid, pyridoxin, riboflavin, thiamine, Cu, Fe, K, Mg, Mn, P, Zn, Ca, Se, and starch fractions). *Abbreviations: RS* = *resistant starch; DC* = *digestible carbohydrates; PGI* = *predicted glycemic index; MCAP* = *medium-chain amylopectin; AM* = *amylose. (C*) Bar plot of color values differences based on CIE Lab*, with fortified rice showing lower lightness and higher a*, b*, and chroma values, consistent with its more saturated golden appearance.

Fortified rice produced under the optimized conditions showed clear improvements in nutritional properties compared to non-fortified rice and changes in appearance (Fig. 2A, and Supplementary Fig. 1). No extensive macro-cracking or structural fragmentation was visually observed under the selected drying conditions. The fortified rice contained substantially higher levels of iron (2802.427 ± 31.254 µg/g; ∼407-fold increase), zinc (813.047 ± 14.248 µg/g; ∼61-fold increase), and thiamine (371.980 ± 13.920 µg/g; ∼413-fold increase) relative to the non-fortified control, while folic acid increased from 1.868 ± 0.214 µg/g to 15.728 ± 0.316 µg/g (∼8.4-fold increase) (Fig. 2B). These enhancements reflect the ability of the ultrasound-assisted soaking process to promote diffusion of micronutrients into the rice grain [24], [25]. It was also observed that minerals such as iron and zinc increased, indicating effective mineral fortification, while protein, lipid, and carbohydrate contents remained within acceptable ranges [26] (Supplementary Table 1).

In terms of color measurements, the fortified rice exhibited a lower lightness value (L* = 64.14 ± 1.21) compared with non-fortified rice (79.87 ± 1.25), alongside higher redness (a* = 4.72 ± 0.22 vs. 1.85 ± 0.11) and yellowness (b* = 20.52 ± 1.62 vs. 13.02 ± 0.59). Chroma increased from 13.16 ± 0.58 to 21.06 ± 1.60, indicating a more saturated golden color, while the hue angle decreased slightly (1.34 ± 0.01 vs. 1.43 ± 0.01) (Fig. 2A and C). These color changes correspond with visual observations, where the fortified rice appeared more yellowish due to the presence of folic acid absorbed during processing [27]. Importantly, despite these changes, the fortified rice maintained an appearance consistent with commonly accepted fortified rice products [28].

The fortified rice is designed as a nutrient-dense concentrate to be blended with ordinary white rice, rather than consumed directly. A 3-g serving of the fortified premix provides approximately 8.41 mg iron (46.7% DV), 0.88 mg bioaccessible thiamine (73% DV), and 0.86 mg bioaccessible zinc (7.8% DV) based on intestinal bioaccessibility estimates. These micronutrient contributions remain well below the established tolerable upper intake levels (ULs) for adults (45 mg/day for iron, 40 mg/day for zinc, and 1000 µg/day for folic acid), indicating that the proposed serving size does not pose a risk of exceeding recognized safety thresholds (Supplementary Table 6). For individuals consuming approximately 2 cups of cooked rice daily (equivalent to roughly 100 g uncooked rice), adding just one 3-g portion of this fortified premix would supply nearly half of the daily iron requirement and almost the full daily requirement for thiamine, without requiring any change in eating behavior or rice preparation methods [29], [30]. This approach provides a practical means of improving nutrient intake in rice-consuming populations and in settings where access to micronutrient-rich foods is limited.

### 3.3 Bioaccessibility assessment of minerals and vitamin B in the fortified rice through simulated digestion revealed Nutrient-Specific digestive stability

**Fig. 3:**
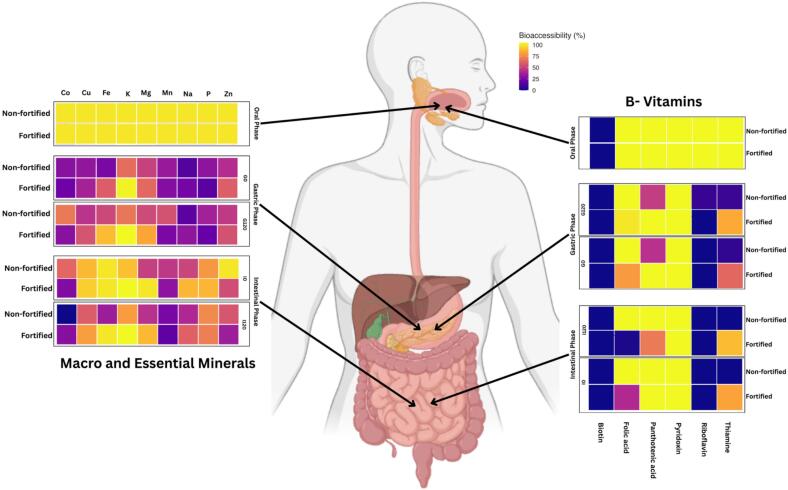
Bioaccessibility of B-vitamins and minerals in fortified and non-fortified rice matrix.

The bioaccessibility results demonstrated that increases in nutrient content do not necessarily translate directly into enhanced physiological availability during digestion (Table 1 and Fig. 3). Thiamine showed the most favorable outcome, with fortified rice maintaining 79% intestinal bioaccessibility (from 371.98 µg/g initially to 293.61 µg/g in the intestinal phase), whereas the non-fortified rice exhibited 0% intestinal bioaccessibility, dropping from 0.90 µg/g to 0.00 µg/g. This suggests that thiamine incorporated through fortification may exist in more soluble and accessible forms than the native thiamine embedded within the rice matrix [14], [31]. Pyridoxin also demonstrated complete bioaccessibility (100%) across all digestion phases, maintaining its initial concentration of 0.34 µg/g, consistent with its known stability under gastrointestinal conditions [32], [33].

**Table 1:** Concentration and bioaccessibility of macro and essential minerals and B-vitamins in fortified and non-fortified rice matrix.

**Fortified**										
**B-vitamins**	**Initial/Oral phase**		**G0**		**G120**		**I0**		**I120**	
	**Total concentration, μg/g**	**Bioaccessibility, %**	**Concentration, μg/g**	**Bioaccessibility, %**	**Concentration, μg/g**	**Bioaccessibility, %**	**Concentration, μg/g**	**Bioaccessibility, %**	**Concentration, μg/g**	**Bioaccessibility, %**
**Thiamine**	371.98 ± 13.92^aA^	100.00 ± 0.00^a^	301.33 ± 5.73^bA^	81.01 ± 1.54^bA^	226.25 ± 13.99^cA^	60.82 ± 3.76^cA^	316.81 ± 13.02^bA^	85.17 ± 3.50^bA^	293.61 ± 2.79^bA^	78.93 ± 0.75^bA^
**Pyridoxin**	0.34 ± 0.00 ^a^	100.00 ± 0.00^a^	0.34 ± 0.00^a^	100.00 ± 0.00^a^	0.34 ± 0.00^a^	100.00 ± 0.00^a^	0.34 ± 0.00^a^	100.00 ± 0.00^a^	0.34 ± 0.00^a^	100.00 ± 0.00^a^
**Pantothenic acid**	1.25 ± 0.14^aB^	100.00 ± 0.00^a^	1.25 ± 0.00^aA^	100.00 ± 0.00^aA^	1.25 ± 0.00^aA^	100.00 ± 0.00^aA^	0.83 ± 0.63^aA^	66.67 ± 50.00^aA^	1.25 ± 0.00^a^	100.00 ± 0.00^a^
**Folic acid**	15.73 ± 0.32^aA^	100.00 ± 0.00^a^	15.02 ± 1.07^aA^	95.50 ± 6.82^aA^	12.03 ± 0.61^bA^	76.64 ± 3.86^bB^	0.28 ± 0.83^dA^	1.75 ± 5.25^dB^	6.17 ± 0.54^cA^	39.24 ± 3.42^cB^
**Biotin**	0.00 ± 0.00^a^	−	0.00 ± 0.00^a^	−	0.00 ± 0.00^a^	−	0.00 ± 0.00^a^	−	0.00 ± 0.00^a^	−
**Riboflavin**	0.37 ± 0.01^aA^	100.00 ± 0.00^a^	0.00 ± 0.00^bA^	0.00 ± 0.00^bA^	0.00 ± 0.00^b^	0.00 ± 0.00^b^	0.00 ± 0.00^b^	0.00 ± 0.00^b^	0.00 ± 0.00^b^	0.00 ± 0.00^b^

**Non-Fortified**										

**B-vitamins**	**Initial/Oral phase**		**G0**		**G120**		**I0**		**I120**	
	**Total concentration, μg/g**	**Bioaccessibility, %**	**Concentration, μg/g**	**Bioaccessibility, %**	**Concentration, μg/g**	**Bioaccessibility, %**	**Concentration, μg/g**	**Bioaccessibility, %**	**Concentration, μg/g**	**Bioaccessibility, %**

**Thiamine**	0.90 ± 0.03^aB^	100.00 ± 0.00^a^	0.07 ± 0.03^bB^	7.86 ± 3.43^bB^	0.05 ± 0.07^bB^	5.51 ± 7.87^bB^	0.00 ± 0.00^bB^	0.00 ± 0.00^bB^	0.00 ± 0.00^b^	0.00 ± 0.00^bB^
**Pyridoxin**	0.34 ± 0.00^a^	100.00 ± 0.00^a^	0.34 ± 0.00^a^	100.00 ± 0.00^a^	0.34 ± 0.00^a^	100.00 ± 0.00^a^	0.34 ± 0.00^a^	100.00 ± 0.00^a^	0.34 ± 0.00^a^	100.00 ± 0.00^a^
**Pantothenic acid**	1.77 ± 0.03^aA^	100.00 ± 0.00^a^	0.82 ± 0.11^bB^	46.13 ± 5.99^bB^	0.75 ± 0.13^bB^	42.46 ± 7.48^bB^	1.77 ± 0.00^aA^	100.00 ± 0.00^aA^	1.77 ± 0.00^aB^	100.00 ± 0.00^a^
**Folic acid**	1.87 ± 0.21^aB^	100.00 ± 0.00^a^	1.87 ± 0.00^aB^	100.00 ± 0.00^aA^	1.87 ± 0.00^aB^	100.00 ± 0.00^aA^	1.87 ± 0.00^aA^	100.00 ± 0.00^aA^	1.87 ± 0.00^aB^	100.00 ± 0.00^aA^
**Biotin**	0.00 ± 0.00^a^	−	0.00 ± 0.00^a^	−	0.00 ± 0.00^a^	−	0.00 ± 0.00^a^	−	0.00 ± 0.00^a^	−
**Riboflavin**	0.37 ± 0.01^aA^	100.00 ± 0.00^a^	0.02 ± 0.05^bA^	6.52 ± 13.17^bA^	0.00 ± 0.00^b^	0.00 ± 0.00^b^	0.00 ± 0.00^b^	0.00 ± 0.00^b^	0.00 ± 0.00^b^	0.00 ± 0.00^b^

**Fortified**										

**Mineral**	**Initial phase**		**G0**		**G120**		**I0**		**I120**	

**Total concentration (µg/g)**	**Bioaccessibility (%)**	**Concentration (µg/g)**	**Bioaccessibility (%)**	**Concentration (µg/g)**	**Bioaccessibility (%)**	**Concentration (µg/g)**	**Bioaccessibility (%)**	**Concentration (µg/g)**	**Bioaccessibility (%)**

**Co**	0.08 ± 0.00^aA^	100.00 ± 0.00^aA^	0.02 ± 0.00^bA^	22.09 ± 0.00^eB^	0.02 ± 0.00^bB^	28.90 ± 0.00^bB^	0.02 ± 0.00^bB^	26.86 ± 0.00^dB^	0.02 ± 0.00^bA^	28.11 ± 0.00^cA^
**Cu**	2.32 ± 0.02^aB^	100.00 ± 0.00^aA^	0.87 ± 0.15^dA^	37.46 ± 6.49^dA^	1.32 ± 0.09^cA^	56.80 ± 3.76^cA^	2.17 ± 0.13^aB^	93.53 ± 5.63^aA^	1.88 ± 0.08^bA^	81.11 ± 3.41^bA^
**Fe**	2802.42 ± 31.25^aA^	100.00 ± 0.00^aA^	482.27 ± 3.62^cA^	60.10 ± 0.46^cA^	713.96 ± 3.41^bA^	88.98 ± 0.43^bA^	2802.42 ± 0.00^aA^	100.00 ± 0.00^aA^	2802.42 ± 0.00^aA^	100.00 ± 0.00^aA^
**K**	462.88 ± 2.49^aB^	100.00 ± 0.00^bA^	462.88 ± 0.00^aB^	100.00 ± 0.00^aA^	462.88 ± 0.00^aB^	100.00 ± 0.00^aA^	462.88 ± 0.00^aB^	100.00 ± 0.00^aA^	462.88 ± 0.00^aB^	100.00 ± 0.00^aA^
**Mg**	186.92 ± 1.33^aB^	100.00 ± 0.00^aA^	120.21 ± 2.51^cB^	64.31 ± 1.34^cA^	155.63 ± 10.41^bB^	83.26 ± 5.57^bA^	186.92 ± 0.00^aB^	100.00 ± 0.00^aA^	165.26 ± 17.42^abB^	88.41 ± 9.32^abA^
**Mn**	16.76 ± 0.11^aA^	100.00 ± 0.00^aA^	4.59 ± 0.04^bcA^	27.37 ± 0.24^bcB^	4.20 ± 0.03^bcB^	25.06 ± 0.20^bcB^	5.06 ± 1.29^bA^	30.17 ± 7.73^bA^	3.02 ± 0.31^cA^	18.03 ± 1.85^cB^
**Na**	8896.22 ± 2815.28^aA^	100.00 ± 0.00^aA^	2113.19 ± 23.31^cB^	23.75 ± 0.26^eA^	2744.60 ± 27.56^cB^	30.85 ± 0.31^dA^	7793.18 ± 38.35^abB^	87.60 ± 0.43^bA^	5485.45 ± 35.94^bcB^	61.66 ± 0.41^cA^
**P**	1106.07 ± 9.88^aB^	100.00 ± 0.00^aA^	180.21 ± 9.34^eB^	16.29 ± 0.84^eB^	221.05 ± 5.59^dB^	19.98 ± 0.51^dB^	967.48 ± 13.31^bB^	87.47 ± 1.21^bA^	784.77 ± 5.71^cB^	70.95 ± 0.51^cB^
**Zn**	813.05 ± 14.25^aA^	100.00 ± 0.00^aA^	491.65 ± 3.08^bA^	60.47 ± 0.38^bA^	454.29 ± 2.89^cA^	55.87 ± 0.36^cA^	441.49 ± 1.64^cA^	54.30 ± 0.20^dB^	287.37 ± 0.92^dA^	35.34 ± 0.11^eB^

**Non-fortified**										

**Mineral**	**Initial phase**		**G0**		**G120**		**I0**		**I120**	

**Total concentration (µg/g)**	**Bioaccessibility (%)**	**Concentration (µg/g)**	**Bioaccessibility (%)**	**Concentration (µg/g)**	**Bioaccessibility (%)**	**Concentration (µg/g)**	**Bioaccessibility (%)**	**Concentration (µg/g)**	**Bioaccessibility (%)**

**Co**	0.04 ± 0.00^aB^	100.00 ± 0.00^aA^	0.01 ± 0.00^cB^	28.85 ± 0.00^dA^	0.03 ± 0.00^bA^	69.48 ± 0.00^bA^	0.03 ± 0.00^bA^	63.46 ± 0.00^cA^	0.00 ± 0.00^dB^	0.00 ± 0.00^eB^
**Cu**	2.91 ± 0.05^aA^	100.00 ± 0.00^aA^	0.91 ± 0.02^eA^	31.32 ± 0.80^eA^	1.31 ± 0.13^dA^	44.88 ± 4.55^dB^	2.57 ± 0.15^bA^	88.10 ± 5.30^bA^	1.74 ± 0.05^cA^	59.84 ± 1.67^cB^
**Fe**	6.89 ± 0.03^aB^	100.00 ± 0.00^aA^	1.72 ± 0.15^dB^	24.93 ± 2.14^dB^	3.61 ± 0.34^bB^	52.35 ± 4.86^bB^	6.89 ± 0.00^aB^	100.00 ± 0.00^aA^	2.41 ± 0.23^cB^	34.99 ± 3.42^cB^
**K**	1109.64 ± 25.90^aA^	100.00 ± 0.00^aA^	726.57 ± 8.19^dA^	65.48 ± 0.74^eB^	755.99 ± 10.30^dA^	68.13 ± 0.93^dB^	998.29 ± 5.64^bA^	89.97 ± 0.50^bB^	893.64 ± 3.83^cA^	80.53 ± 0.35^cB^
**Mg**	547.22 ± 11.98^aA^	100.00 ± 0.00^aA^	273.74 ± 7.13^bcA^	50.02 ± 1.30^bB^	291.73 ± 1.83^bA^	53.31 ± 0.34^bB^	271.18 ± 23.15^bcA^	49.55 ± 4.23^bcB^	242.35 ± 1.53^cA^	44.29 ± 0.28^cB^
**Mn**	12.78 ± 0.33^aB^	100.00 ± 0.00^aA^	4.28 ± 0.19^cdA^	33.46 ± 1.52^cdA^	7.13 ± 0.67^bA^	55.81 ± 5.25^bA^	5.97 ± 1.53^bcA^	46.74 ± 11.98^bcA^	3.13 ± 0.02^dA^	24.46 ± 0.13^dA^
**Na**	17902.71 ± 13376.24^aA^	100.00 ± 0.00^aA^	2609.70 ± 43.38^aA^	14.58 ± 0.25^eB^	3065.50 ± 10.65^aA^	17.13 ± 0.06^dB^	9207.94 ± 45.32^aA^	51.43 ± 0.26^bB^	7258.84 ± 38.06^aA^	40.54 ± 0.21^cB^
**P**	1450.64 ± 34.16^aA^	100.00 ± 0.00^aA^	438.36 ± 6.17^eA^	30.22 ± 0.43^eA^	533.88 ± 7.88^dA^	36.80 ± 0.55^dA^	1143.12 ± 15.72^bA^	78.80 ± 1.09^bB^	1082.91 ± 19.33^cA^	74.65 ± 1.33^cA^
**Zn**	13.23 ± 0.14^aB^	100.00 ± 0.00^aA^	5.64 ± 0.23^dB^	42.57 ± 1.76^dB^	6.21 ± 0.11^cB^	46.93 ± 0.83^cB^	13.23 ± 0.00^aB^	100.00 ± 0.00^aA^	7.59 ± 0.14^bB^	57.35 ± 1.07^bA^

Data are presented as mean ± standard deviation (n = 3). Means within each dependent measure were analyzed using one-way ANOVA followed by Tukey's HSD post-hoc test. Values with different capital letters within the same row indicate significant differences between fortified and non-fortified samples at the same digestive phase. Values with different small letters within the same column indicate significant differences across digestive phases for the same treatment. Significance was set at p < 0.05.

In contrast, folic acid exhibited a marked decline during digestion. Although fortified rice retained 76.64% bioaccessibility during the gastric phase, intestinal bioaccessibility decreased to 39.24%, with the intestinal concentration dropping to 6.17 µg/g. This reduction is consistent with the pH-dependent instability of folate, its reduced solubility at different pH, and potential binding to soluble fiber released from the rice during digestion [32], [33], [34]. Riboflavin bioaccessibility was below the detection limit of the analytical method in both fortified and non-fortified rice, declining from 0.37 µg/g at the initial phase to 0.00 µg/g in the intestinal phase, aligning with its known susceptibility to degradation during cooking and digestion [32], [33]. Given that only 39.24% of fortified folic acid remained intestinally bioaccessible, further optimization of premix levels may be necessary following in vivo validation.

Fortified rice maintained complete intestinal bioaccessibility of iron (100%) and moderate retention of zinc (35.34%) during the intestinal phase. Both minerals remained fully soluble under oral and gastric conditions (100% bioaccessibility). While mineral solubility in the intestinal environment can be influenced by pH and matrix interactions, the present results demonstrate sustained mineral recovery in the soluble fraction under the applied in vitro digestion conditions. The sustained recovery of iron in the soluble fraction suggests effective retention under the applied digestion conditions, although speciation and absorption efficiency were not determined, whereas the partial reduction observed for zinc may reflect pH-dependent complex formation in the intestinal environment [32], [33], [35], [36]. While the specific mechanisms were not investigated in this study, the observed behavior differs from commonly reported mineral precipitation patterns in cereal matrices [37], [38]. These findings demonstrate that ultrasonication-assisted fortification can enhance mineral retention during digestion, although dedicated bioavailability studies remain necessary [[35], [36], [37], [38], [39]]. Nevertheless, dedicated in vivo bioavailability studies are warranted to confirm physiological mineral absorption.

When the intestinally bioaccessible fraction was compared with recommended dietary allowances (RDA), the fortified rice retained substantial levels of bioaccessible thiamine and pyridoxine, contributing meaningfully to daily vitamin requirements. Based on the estimated intestinally bioaccessible fraction, the fortified product could provide approximately 46.7% of the daily value (DV) for iron and 7.8% DV for zinc per 3-g serving, although dedicated bioavailability assessments are required to validate these estimates.

### 3.4 Ultrasonication not only influenced the fortificants but also the bioactive compounds

**Fig. 4:**
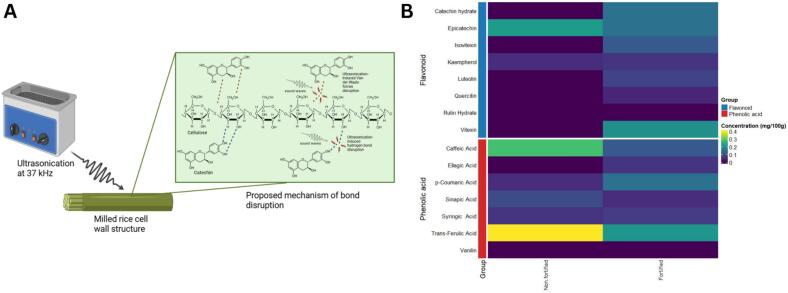
Mechanistic insight and phenolic profile shifts induced by ultrasonication. (A) Proposed mechanism for the bond disruption during ultrasonication (B) Complex heatmap diagram of the concentrations of selected phenolic compounds after ultrasonication.

In this study, soaking the grains in an aqueous solution containing the fortificants, combined with ultrasonic treatment resulted in fortified rice exhibiting higher levels of phenolic compounds such as catechin hydrate (0.15 ± 0.01 ug/g), ellagic acid (0.06 ± 0.00 ug/g), luteolin (0.08 ± 0.00 ug/g), Isovitexin (0.11 ± 0.02 ug/g), p-Coumaric acid (0.15 ± 0.01 ug/g), Quercitin (0.04 ± 0.01 ug/g), Syringic Acid (0.07 ± 0.00 ug/g), and Vitexin (0.20 ± 0.03 ug/g) compared to non-fortified rice (Table 2 and Fig. 4). These flavonoids are inherently unstable because of their structural properties, and their concentrations increased after ultrasonic treatment. The C6–C3–C6 backbone structure of flavonoids, characterized by multiple hydroxyl groups [40], [41], facilitates their adsorption onto cellulose components of rice cell walls through van der Waals interactions and hydrogen bonding [42]. These non-covalent interactions may become destabilized under cavitation-induced shear forces during ultrasonication (Fig. 4A), potentially promoting bond loosening, partial glycosidic cleavage, and enhanced release of flavonoids from the matrix. Their planar conjugated aromatic structures may facilitate their release from bound forms within the cell wall matrix, thereby enhancing their incorporation and detectability after fortification [[43], [44]]. Furthermore, because their hydroxyl groups are attached to rigid aromatic rings, their solubility in highly polar solvents such as water is limited, reducing their likelihood of being leached during the soaking step.

**Table 2:** Concentration of selected phenolic compounds, TVA, RVA, and color parameters for fortified and non-fortified rice.

Phenolic Compounds, mg/100 g	Non-fortified	Fortified
Caffeic Acid	0.28 ± 0.01^a^	0.11 ± 0.02^b^
Catechin hydrate	0.00 ± 0.00^b^	0.15 ± 0.01^a^
Ellagic Acid	0.00 ± 0.00^b^	0.06 ± 0.00^a^
Epicatechin	0.22 ± 0.03^a^	0.15 ± 0.01^b^
Isovitexin	0.00 ± 0.00^b^	0.11 ± 0.02^a^
Kaempherol	0.05 ± 0.00^a^	0.06 ± 0.00^a^
Luteolin	0.00 ± 0.00^b^	0.08 ± 0.00^a^
p-Coumaric Acid	0.05 ± 0.00^b^	0.15 ± 0.01^a^
Quercitin	0.00 ± 0.00^b^	0.04 ± 0.01^a^
Rutin Hydrate	0.00 ± 0.00^a^	0.00 ± 0.00^a^
Sinapic Acid	0.09 ± 0.02^a^	0.05 ± 0.01^a^
Syringic Acid	0.06 ± 0.00^a^	0.07 ± 0.00^a^
Trans-Ferulic Acid	0.40 ± 0.01^aa^	0.21 ± 0.01^b^
Vanilin	0.00 ± 0.00^a^	0.00 ± 0.00^a^
Vitexin	0.00 ± 0.00^b^	0.20 ± 0.03^a^

**TPA parameters**		
Hardness, g	1858.63 ± 29.45^a^	1240.47 ± 124.84^a^
Adhesiveness, g.sec	8.23 ± 1.26^a^	8.56 ± 1.59^a^
Cohesiveness	0.53 ± 0.01^a^	0.36 ± 0.03^a^
Springiness	0.08 ± 0.00^a^	0.05 ± 0.01^a^
Chewiness	76.43 ± 4.14^a^	23.23 ± 1.45^b^

**RVA Parameters**		
Peak viscosity, cP	3463.00 ± 16.00^a^	1953.00 ± 18.00^b^
Trough viscosity, cP	2758.00 ± 18.00^a^	983.00 ± 11.00^b^
Breakdown viscosity, cP	705.00 ± 2.00^b^	971.00 ± 8.00^a^
Final viscosity, cP	5948.00 ± 54.00^a^	1468.00 ± 23.00^b^
Setback viscosity, cP	3190.00 ± 35.00^a^	485.00 ± 13.00^b^
Peak time, min	5.87 ± 0.00^a^	5.57 ± 0.05^a^
Pasting temperature, deg C	86.48 ± 0.11^b^	87.25 ± 0.07^a^

**Color parameters**		
L*	64.14 ± 1.21^b^	79.87 ± 1.25^a^
a*	4.72 ± 0.22^a^	1.85 ± 0.11^b^
b*	20.52 ± 1.62^a^	13.02 ± 0.59^b^
Chroma	21.06 ± 1.60^a^	13.16 ± 0.58^b^
Hue angle	1.34 ± 0.01^b^	1.43 ± 0.01^a^

Data are presented as mean ± standard deviation (n = 3) for phenolic compounds and (n = 2) for TPA, RVA, and color parameters. Within each dependent measure, means within different subscripts differ significantly (p < 0.05).

After fortification, several phenolic acids decreased in concentration, including *trans*-ferulic acid (0.21 ± 0.01 µg/g; ∼1.90-fold decrease), epicatechin (0.15 ± 0.01 µg/g; ∼1.47-fold decrease), caffeic acid (0.11 ± 0.02 µg/g; ∼2.55-fold decrease), and sinapic acid (0.05 ± 0.01 µg/g; ∼1.80-fold decrease) (Fig. 4B). Several of these compounds belong to the cinnamic and benzoic acid families, whose –OH and –COOH functional groups confer high aqueous solubility, making them more susceptible to leaching during the soaking step. The presence of ionizable carboxyl groups promotes dissolution in water, which likely contributed to their reduced levels. This suggests that ultrasonication may affect the stability or extractability of more labile phenolic acids while simultaneously favoring the release, migration, or retention of flavonoids. This response divergence is in line with the effects of acoustic cavitation, which has been shown to disturb cell wall matrices and encourage the release and alteration of phenolic constituents [45], [46], [47].

Other factors, including cell wall restructuring, compound stability, and changes in phenolic bioavailability during cavitation, may also contribute and cannot be excluded without targeted experiments. Thus, the present findings should be regarded as a proposed mechanism rather than conclusive evidence. Further validation through controlled leaching tests, cellulose–phenolic binding assays, and/or molecular dynamics simulations is needed to clarify how ultrasonication alters polyphenol conformation and interactions with the starchy endosperm, including whether specific non-covalent bonds are preferentially disrupted.

### 3.5 Effects of ultrasonication on the physicochemical and textural properties of fortified rice

**Fig. 5:**
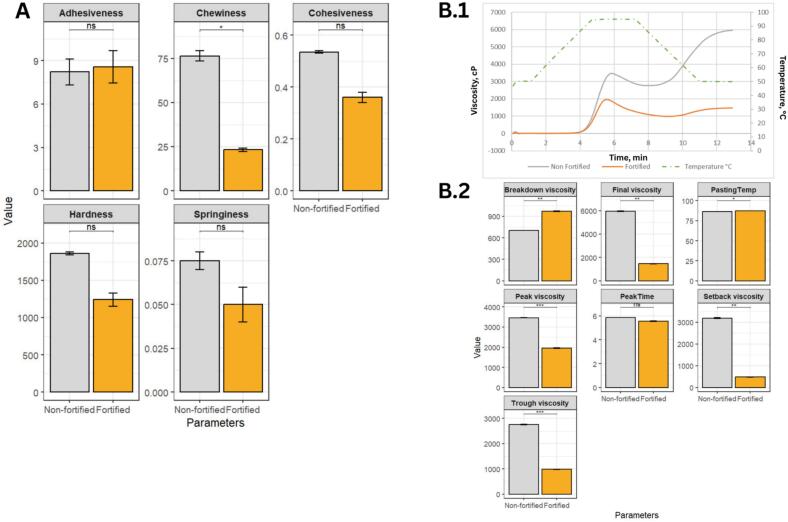
Textural and rheological profile analysis of fortified and non-fortified rice. (A) Barplot of the texture profile analysis of fortified and non-fortified samples. (B.1) Rapid visco analyzer (RVA) curve of fortified and non-fortified rice. (B.2) Barplot of the rheological behavior and viscosity profiles of fortified vs. non-fortified samples.

Textural Profile Analysis of fortified and non-fortified rice (Fig. 5A) revealed no significant differences in adhesiveness, cohesiveness, or springiness between the two samples. Although hardness was also not statistically different, its marked reduction in value suggests a tendency toward softer grain structure following fortification. In contrast, chewiness decreased significantly, indicating a pronounced shift in the mechanical resistance of the fortified rice during mastication. It should be noted that the present textural evaluation was conducted to assess whether the ultrasonication-assisted fortification process induces measurable kernel-level changes in texture-related properties, rather than to predict the sensory characteristics of a blended consumer product where fortified kernels may be incorporated at low proportions. Thus, the observed texture differences are interpreted as process-driven effects of ultrasonication (e.g., matrix weakening and/or partial leaching of soluble components during cavitation), rather than solely the result of mixing a small number of fortified grains with bulk rice. This divergence in chewiness may be attributed to differences in starch composition, as the present findings contrast with those of Bonto et al. (2024) [48], who reported the opposite trend in waxy, low-amylose rice. The discrepancy is likely driven by compositional differences, as the present study utilized non-waxy, high-amylose low-GI rice. High-amylose rice possesses more linear chains that are structurally less stable under acoustic cavitation. Prior work has shown that ultrasonication preferentially disrupts these low-integrity amylose regions [49], which may explain the reduction in chewiness observed in the fortified sample. Overall, the results suggest that ultrasonication can be applied without major adverse effects on key textural attributes, while potentially lowering chewiness and mastication resistance.

Ultrasonic fortification also significantly influenced the physicochemical and pasting behavior of rice starch, indicating structural and molecular reconfigurations resulting from acoustic cavitation. As shown in Fig. 5B.1 and 5B.2, there is a pronounced decrease in peak viscosity (3463 → 1953 cP), trough viscosity (2758 → 983 cP), and final viscosity (5948 → 1468 cP) which suggests that the high-intensity ultrasonic waves disrupted starch granule integrity and partially degraded the amylopectin matrix [50], [51], [52]. Moreover, the cavitational forces generated during ultrasonication can enhance granule porosity, as the formation of pores, channels, and cavities increases the specific surface area and facilitates the diffusion of reagents and enzymes into the starch matrix [49]. This structural modification may accelerate enzymatic hydrolysis [53] and ultimately improve nutrient bioavailability. The sharp decline in setback viscosity (3190 → 485 cP) reflects a substantial reduction in the retrogradation tendency of the fortified rice starch, which can be attributed to the inhibited reassociation of amylose chains following ultrasonic-induced disruption of the granular structure. These rheological shifts corresponded with alterations in starch fractions, including reduced AM1 and short-chain amylopectin fractions, alongside increased AM2 (Fig. 2).This observation is consistent with reports on faba bean starch, where ultrasonication decreased the rate of retrogradation depending on the degree of molecular reassociation and the molecular weight and chain length of starch components [54]. Comparable trends have also been observed in potato starch pastes, wherein lower ultrasonic power densities effectively reduced retrogradation due to partial depolymerization and the consequent alteration of intermolecular interactions [55]. A slight increase in pasting temperature (86.48 → 87.25°C) indicates that ultrasonication may have promoted subtle molecular rearrangements within the starch matrix [56], [57]. These physicochemical and textural modifications indicate functional shifts that may be relevant to applications such as ready-to-eat (RTE) or convenience-type rice products, where reduced chewiness, softer grain attributes, and lower viscosity profiles are often associated with improved handling and processing behavior. The observed decrease in retrogradation tendency further suggests altered structural stability during storage, a characteristic commonly linked with products subjected to refrigeration or reheating cycles.

## 4 Conclusion

This study demonstrates that ultrasonication-assisted processing provides a rapid and scalable strategy for multi-micronutrient fortification of polished rice while maintaining key physicochemical and functional properties. Process optimization using RSM identified 2.734 min sonication, 4999.99 ppm fortificant concentration, and 240 min soaking as optimal conditions to maximize the uptake and incorporation of iron, zinc, thiamine, and folic acid. Under these conditions, fortified kernels showed substantial enrichment of iron (2802.42 ± 31.25 µg/g; ∼407-fold), zinc (813.05 ± 14.25 µg/g; 61-fold), thiamine (371.98 ± 13.92 µg/g; ∼413-fold), and folic acid (15.73 ± 0.32 µg/g; ∼8-fold). A 3-g serving of fortified rice premix can provide approximately 8.41 mg iron (46.7% DV), 0.88 mg bioaccessible thiamine (73% DV), and 0.86 mg bioaccessible zinc (7.8% DV) based on intestinal bioaccessibility estimates, supporting its use as a nutrient-dense concentrate for blending with regular rice. Importantly, these micronutrient contributions remain substantially below established tolerable upper intake levels (ULs), confirming that the proposed premix serving size remains within recognized safety thresholds. In vitro digestion revealed nutrient-specific bioaccessibility, with fortified rice retaining 79% intestinal bioaccessibility of thiamine, while folic acid showed reduced intestinal bioaccessibility (39.24%), indicating that in vivo validation and blending ratio refinement are warranted. Notably, fortified rice maintained complete intestinal bioaccessibility of iron (100%) and moderate retention of zinc (35.34%), highlighting effective mineral stabilization during ultrasonication. Ultrasonication altered the phenolic profile, increasing several flavonoids while reducing certain soluble phenolic acids (e.g., ferulic, caffeic, and sinapic acids), suggesting trade-offs likely linked to cavitation-driven release and leaching. Importantly, key eating-quality parameters were largely maintained, with no significant changes in adhesiveness, cohesiveness, or springiness, and a significant reduction in chewiness, indicating potential improvements in mastication resistance. Ultrasonication also reduced RVA viscosity and setback values, implying modified starch structure and lower retrogradation tendency, which may be advantageous for processed and convenience rice products. Overall, ultrasonication-assisted fortification provides a scalable, energy-efficient platform for producing multi-micronutrient fortified rice kernels that can support value-added food formulations and public health fortification programs aimed at reducing hidden hunger.

## Funding declaration

This research was funded by the Department of Agriculture and Farmers Welfare (DA&FW) and the Indian Council of Agricultural Research (ICAR-IRRI work plan 2024–2027).

## CRediT authorship contribution statement

**Glenn Vincent P. Ong:** Writing – review & editing, Writing – original draft, Visualization, Formal analysis, Data curation, Conceptualization. **Iegem Lean Laudencia:** Writing – review & editing, Writing – original draft, Visualization, Formal analysis, Data curation, Conceptualization. **Rhowell Tiozon:** Writing – review & editing, Supervision. **Jhaymes Khylle Jose:** Methodology, Conceptualization. **Vipin Pratap:** Methodology, Conceptualization, Conceptualization. **Nese Sreenivasulu:** Writing – review & editing, Supervision, Project administration, Funding acquisition, Conceptualization.

## Declaration of competing interest

The authors declare that they have no known competing financial interests or personal relationships that could have appeared to influence the work reported in this paper.

## Data Availability

Data will be given upon request.
