# Supplementary figures and images for: Ultrasonication-mediated multi-micronutrient fortification of polished rice to address micronutrient deficiencies
Source: Ultrason Sonochem. 2026 Mar 10;128:107814. doi: 10.1016/j.ultsonch.2026.107814 (PMC13000705; doi:10.1016/j.ultsonch.2026.107814)

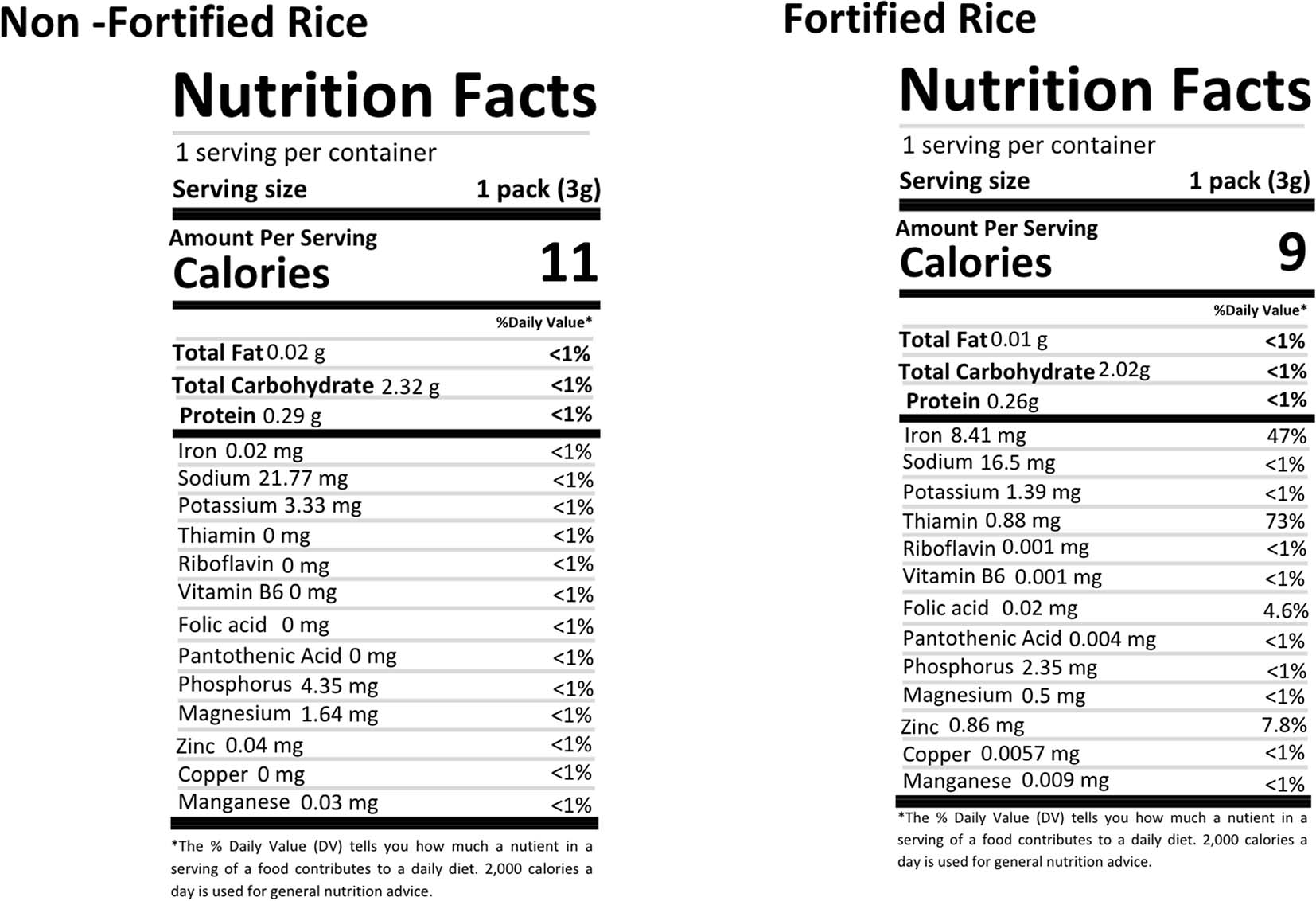

Supplement: Supplementary Fig. 1 [file mmc1.jpg]
