# Supplementary material for: Ultrasonication-mediated multi-micronutrient fortification of polished rice to address micronutrient deficiencies
Source: Ultrason Sonochem. 2026 Mar 10;128:107814. doi: 10.1016/j.ultsonch.2026.107814 (PMC13000705; doi:10.1016/j.ultsonch.2026.107814)
Supplement: Supplementary Data 1 [file mmc2.docx]

**Supplementary Table 1.** Concentration of vitamins and minerals of Low GI 108 in different fortification parameters.

| Treatment | Sonication | Concentration | Soaking | Vitamin B1 (μg/g) | Vitamin B9 (μg/g) | Iron (μg/g) | Zinc (μg/g) |
| --- | --- | --- | --- | --- | --- | --- | --- |
| 1 | 7.5 | 3000 | 135 | 1150.28 ± 57.73^ef^ | 14.27 ± 2.89^d^ | 1428.28 ± 4.16^gh^ | 341.94 ± 34.27^fgh^ |
| 2 | 7.5 | 5000 | 240 | 1697.28 ± 31.02^b^ | 8.82 ± 0.00^d^ | 2469.10 ± 26.89^b^ | 675.62 ± 33.25^b^ |
| 3 | 7.5 | 1000 | 30 | 560.47 ± 70.15^h^ | 186.98 ± 15.72^a^ | 370.96 ± 23.44^j^ | 207.16 ± 16.09^i^ |
| 4 | 7.5 | 5000 | 30 | 1405.03 ± 53.20^c^ | 47.94 ± 12.58^c^ | 1912.88 ± 12.22^d^ | 586.67 ± 24.52^cd^ |
| 5 | 15 | 3000 | 240 | 1260.26 ± 47.35^cd^ | 8.90 ± 0.58^d^ | 1641.41 ± 38.50^e^ | 426.44 ± 28.48^ef^ |
| 6 | 0 | 3000 | 30 | 1607.10 ± 54.56^b^ | 18.70 ± 1.53^d^ | 1893.57 ± 30.86^d^ | 631.55 ± 15.39^bc^ |
| 7 | 15 | 1000 | 135 | 595.54 ± 81.17^h^ | 99.74 ± 7.02^b^ | 298.85 ± 21.50^jk^ | 262.57 ± 27.54^hi^ |
| 8 | 0 | 5000 | 135 | 1888.53 ± 28.57^a^ | 2.58 ± 0.00^d^ | 3134.61 ± 18.93^a^ | 937.96 ± 20.21^a^ |
| 9 | 15 | 3000 | 30 | 1201.47 ± 40.36^def^ | 21.94 ± 0.00^d^ | 1370.31 ± 44.02^h^ | 427.54 ± 13.80^e^ |
| 10 | 15 | 5000 | 135 | 1345.37 ± 66.20^cd^ | 8.85 ± 1.73^d^ | 2269.74 ± 12.70^c^ | 526.17 ± 32.53^d^ |
| 11 | 0 | 3000 | 240 | 1615.31 ± 52.55^b^ | 11.26 ± 4.51^d^ | 2411.54 ± 23.26^b^ | 678.87 ± 25.94^b^ |
| 12 | 7.5 | 3000 | 135 | 1162.20 ± 88.56^ef^ | 10.91 ± 1.15^d^ | 1464.58 ± 10.58^g^ | 408.69 ± 16.04^efg^ |
| 13 | 0 | 1000 | 135 | 843.10 ± 56.71^g^ | 102.21 ± 8.50^b^ | 498.82 ± 43.55^i^ | 333.33 ± 27.65^gh^ |
| 14 | 7.5 | 3000 | 135 | 1045.61 ± 71.04^f^ | 21.69 ± 2.08^d^ | 1543.03 ± 9.50^f^ | 378.66 ± 45.01^efg^ |
| 15 | 7.5 | 1000 | 240 | 683.69 ± 53.00^gh^ | 84.43 ± 12.22^b^ | 291.94 ± 33.25^k^ | 270.32 ± 35.00^hi^ |
| 16 | 7.5 | 3000 | 135 | 1063.95 ± 67.51^f^ | 9.40 ± 2.31^d^ | 1443.13 ± 20.22^gh^ | 371.42 ± 23.64^efg^ |
| 17 | 7.5 | 3000 | 135 | 1094.38 ± 60.58^ef^ | 7.73 ± 0.58^d^ | 1463.93 ± 10.21^g^ | 405.53 ± 33.65^efg^ |

Data are presented as mean ± standard deviation (n = 3). Means within each nutrient (Vitamin B1, Vitamin B9, Iron, and Zinc) were analyzed using one-way analysis of variance (ANOVA) followed by Tukey’s HSD post-hoc test. Values with different lowercase letters within the same column indicate statistically significant differences among treatments (p < 0.05).

**Supplementary Table 2.** Analysis of variance (ANOVA) and model statistics for the response surface models.

| **p-value** | **Vitamin B1** | | **Vitamin B9** | | **Iron** | | **Zinc** | |
| --- | --- | --- | --- | --- | --- | --- | --- | --- |
| Model | < 0.0001 | | 0.0009 | | < 0.0001 | | < 0.0001 | |
| A - Sonication | < 0.0001 | | 0.9251 | | 0.0004 | | < 0.0001 | |
| B - Concentration | < 0.0001 | | < 0.0001 | | < 0.0001 | | < 0.0001 | |
| C - Soaking | 0.0274 | | 0.0120 | | 0.0123 | | 0.0388 | |
| AB | 0.0471 | | 0.8050 | | 0.0417 | | 0.0005 | |
| AC | 0.6930 | | 0.8741 | | 0.3864 | | 0.4101 | |
| BC | 0.2113 | | 0.1050 | | 0.0491 | | 0.6551 | |
| A^2^ | 0.0003 | | 0.1573 | | 0.0016 | | < 0.0001 | |
| B^2^ | 0.0028 | | 0.0003 | | 0.0073 | | 0.3397 | |
| C^2^ | 0.0056 | | 0.1033 | | 0.6010 | | 0.0211 | |
| Lack of Fit | 0.2593 | | 0.0066 | | 0.0072 | | 0.4619 | |
| **MODEL SUMMARY** | |  | |  | |  | |  |
| Mean | 1189.39 | | 39.20 | | 1523.92 | | 462.97 | |
| Standard Deviation | 61.43 | | 17.04 | | 133.64 | | 27.64 | |
| C.V. % | 5.17 | | 43.47 | | 8.77 | | 5.97 | |
| R^2^ | 0.9888 | | 0.9502 | | 0.9882 | | 0.9905 | |
| Adequate Precision | 27.616 | | 13.370 | | 27.632 | | 33.696 | |

*P-values were obtained from analysis of variance (ANOVA) of the response surface models. Terms with p < 0.05 were considered statistically significant. A = sonication time, B = fortificant concentration, and C = soaking time.*

**Supplementary Table 3**. Constraints and numerical output for the optimization of the fortification process.

| **VARIABLE** | **GOAL** | **LOWER LIMIT** | **UPPER LIMIT** |
| --- | --- | --- | --- |
| Sonication | is in range | 0 | 15 |
| Concentration | is in range | 1000 | 5000 |
| Soaking | is in range | 30 | 240 |
| Vitamin B1 | maximize | 560.47 | 1888.53 |
| Vitamin B9 | maximize | 2.58 | 186.98 |
| Iron | maximize | 291.94 | 3134.61 |
| Zinc | maximize | 207.16 | 937.96 |

Optimization constraints and response goals used for numerical optimization of the fortification process. Sonication time (A), concentration (B), and soaking time (C) were set within the experimental ranges, while micronutrient responses were set to maximize.

**Supplementary Table 4**. Summary of equations in terms of coded factors for different responses.

| **RESPONSES** | **EQUATION** |
| --- | --- |
| Vitamin B1 | +1103.28 -193.93A +456.68B +60.31C -73.90AB +12.64AC +42.26BC +199.63A^2^ -134.78B^2^ +118.12C^2^ |
| Vitamin B9 | +12.80 +0.59A -50.65B -20.27C +2.18AB -1.40AC +15.68BC -13.15A^2^ +53.70B^2^ +15.55C^2^ |
| Iron | +1468.59 -294.78A +1040.72B +158.29C -166.22AB -61.72AC +158.81BC +324.95A^2^ -243.04B^2^ +35.66C^2^ |
| Zinc | +381.25 -117.37A +206.63B +24.79 -85.26AB -12.11AC +6.45BC +119.96A^2^ +13.30B^2^ +39.89C^2^ |

*A = sonication time; B = fortificant concentration; C = soaking time. Equations are expressed in terms of coded factors derived from the response surface methodology model.*

**Supplementary Table 5**. Suggested values of sonication time, concentration, and soaking time for the validation of the fortification process.

| **Sonication** | **Concentration** | **Soaking** | **Desirability** |  |
| --- | --- | --- | --- | --- |
| **2.734** | **4999.999** | **240** | **0.542** | **Selected** |
| 2.505 | 4999.998 | 240 | 0.542 | 2 |
| 2.679 | 4972.867 | 240 | 0.533 | 3 |
| 2.636 | 4951.479 | 240 | 0.526 | 4 |
| 0.643 | 4999.998 | 30 | 0.522 | 5 |

**Supplementary Table 6.** Estimated bioaccessible micronutrient intake from fortified rice (3 g serving) relative to dietary reference intakes and tolerable upper intake levels for adults (19–50 years).

| **Nutrient** | **Fortified Rice (µg/g)** | **Intake from 3g of Fortified Rice (mg)** | **Bioaccessibility %** | **Bioaccessible intake (mg)** | **RDA/RNI Adults (19–50 y)** | **Upper Limit (adults)** | **Daily Value (US FDA)** | **%RDA / %DV Contribution** | **Remarks** |
| --- | --- | --- | --- | --- | --- | --- | --- | --- | --- |
| Thiamine | 371.98 | 1.12 | 78.9 | 0.88 | 1.2 mg | – | 1.2 mg | 73.3% / 73.3% | No UL established; intake from fortified rice considered safe |
| Pyridoxine | 0.34 | 0.001 | 100 | 0.001 | 1.3 mg | 100 mg | 1.7 mg | 0.08% / 0.06% | ~99.99% below UL |
| Pantothenic acid | 1.25 | 0.004 | 100 | 0.004 | 5 mg | – | 5 mg | 0.08% / 0.08% | No UL established; intake within safe range |
| Folic acid | 15.73 | 0.047 | 39.2 | 0.02 | 400 µg | 1000 µg | 400 µg DFE | 4.6% / 4.6% | ~98% below UL |
| Biotin | 0 | 0 | – | – | 30 µg | – | 30 µg | – | No UL established; intake considered safe |
| Riboflavin | 0.37 | 0.001 | 0 | 0 | 1.3 mg | – | 1.3 mg | 0% / 0% | No UL established; intake considered safe |
| Co | 0.08 | 0.00024 | 28.1 | 0.00007 | No RDA | – | – | – | No RDA or UL established; trace level detected |
| Cu | 2.32 | 0.007 | 81.1 | 0.0057 | 900 µg | 10 mg | 0.9 mg | 0.63% / 0.63% | ~99.9% below UL |
| Fe | 2802.4 | 8.41 | 100 | 8.41 | 8 mg (men), 18 mg (women) | 45 mg | 18 mg | 105% (men) / 47% (women); 46.7% DV | ~81% below UL |
| K | 462.88 | 1.39 | 100 | 1.39 | 3400 mg | – | 4700 mg | 0.04% / 0.03% | No UL established for potassium from food sources |
| Mg | 186.92 | 0.56 | 88.4 | 0.5 | 420 mg | 350 mg* | 420 mg | 0.12% / 0.12% | >99% below UL; UL applies only to supplemental magnesium |
| Mn | 16.76 | 0.05 | 18 | 0.009 | 2.3 mg | 11 mg | 2.3 mg | 0.39% / 0.39% | ~99.9% below UL |
| Na | 8896.2 | 26.69 | 61.7 | 16.5 | 1500 mg | 2300 mg | 2300 mg | 1.10% / 0.72% | ~99% below UL |
| P | 1106.1 | 3.32 | 71 | 2.35 | 700 mg | 4000 mg | 1250 mg | 0.34% / 0.19% | ~99.9% below UL |
| Zn | 813.05 | 2.44 | 35.3 | 0.86 | 11 mg | 40 mg | 11 mg | 7.8% / 7.8% | ~97.8% below UL |

**Estimated micronutrient intake was calculated based on a 3 g serving of fortified rice using measured concentrations (µg g⁻¹), which were converted to milligrams per serving. Bioaccessible intake was estimated by multiplying the calculated intake by the corresponding bioaccessibility percentage obtained from in vitro gastrointestinal digestion analysis. Percent contribution to Recommended Dietary Allowances (RDA) or Adequate Intakes (AI) and Daily Values (DV) was calculated based on the estimated bioaccessible intake. RDA/AI and Tolerable Upper Intake Levels (UL) for adults aged 19–50 years were obtained from the Dietary Reference Intake (DRI) reports of the Institute of Medicine and the National Academies of Sciences, Engineering, and Medicine. Daily Values (DV) correspond to nutrition labeling standards established by the U.S. Food and Drug Administration. For nutrients without established UL values (e.g., thiamine, riboflavin, pantothenic acid, biotin, and potassium), intake from food sources is considered to present minimal risk under typical dietary conditions. For micronutrients without established RDA values (e.g., cobalt), reference information was consulted from the World Health Organization and Food and Agriculture Organization guidelines.
